# Successful Surgical Embolectomy Following Veno-Arterial Extracorporeal Membrane Oxygenation in a Taxi Driver With High-Risk Pulmonary Embolism: A Case Report

**DOI:** 10.7759/cureus.94611

**Published:** 2025-10-15

**Authors:** Yoh Arita, Kota Takaki, Hironori Orihashi, Katsukiyo Kitabayashi, Nobuyuki Ogasawara

**Affiliations:** 1 Department of Cardiology, Japan Community Healthcare Organization Osaka Hospital, Osaka, JPN; 2 Department of Cardiovascular Surgery, Japan Community Healthcare Organization Osaka Hospital, Osaka, JPN

**Keywords:** pulmonary embolism, shock, surgical embolectomy, taxi driver, va-ecmo

## Abstract

We report a case of a 51-year-old male taxi driver who developed cardiogenic shock due to massive pulmonary embolism (PE) while working. The patient, with a prior history of myocardial infarction, presented with severe hypoxia, hypotension, and electrocardiographic changes mimicking left main coronary artery infarction. Coronary angiography revealed no significant stenosis, while imaging studies confirmed bilateral central PE and deep vein thrombosis. Despite initial anticoagulation and vasopressor therapy, the patient experienced circulatory collapse, requiring cardiopulmonary resuscitation. Veno-arterial extracorporeal membrane oxygenation (VA-ECMO) was promptly initiated, followed by successful surgical embolectomy (SE). The postoperative course was favorable, and the patient was discharged on hospital day 30 with full recovery.

This case highlights the diagnostic challenges in differentiating PE from acute coronary syndromes in patients presenting with shock. It also underscores the value of risk stratification using clinical scores, such as the simplified Pulmonary Embolism Severity Index (sPESI), and cardiac biomarkers. Importantly, the patient’s occupation - marked by prolonged sitting - represents a significant but underrecognized risk factor for venous thromboembolism. In high-risk PE cases unresponsive to medical therapy, early implementation of VA-ECMO and SE can be lifesaving. Awareness of occupational risk factors and prompt multidisciplinary intervention are critical to improving outcomes in similar clinical scenarios.

## Introduction

Acute pulmonary embolism (PE) requires prompt diagnosis and treatment, particularly when accompanied by shock. The basic pathology of acute PE is progressive circulatory and respiratory failure, and the mortality rate within one hour of disease onset is extremely high, particularly in cases of extensive PE [1]. In severe cases, it is necessary to diagnose, manage, and treat circulatory and respiratory failure simultaneously. In recent years, scoring of acute mortality in patients with PE, based on several important factors, has become widespread [2,3]. The simplified Pulmonary Embolism Severity Index (sPESI) consists of only six items related to general patient background and vital signs, and can be easily used at the bedside [4]. The sPESI score has been reported to be useful in predicting 30-day mortality in patients with PE, helping identify low-risk patients for early hospital discharge or home treatment [5,6].

Patients with hemodynamically significant PE need to undergo systemic anticoagulation, systemic thrombolysis, catheter-directed thrombolysis, catheter-based embolectomy, and advanced surgical therapies, such as surgical embolectomy (SE) and mechanical circulatory support. SE and veno-arterial extracorporeal membrane oxygenation (VA-ECMO) are used infrequently, in cases where other treatment modalities have failed [7,8]. The most recent American Heart Association scientific statement indicates that, despite surgical outcomes for severe cases in which preoperative cardiopulmonary resuscitation (CPR) was performed (4.9%-45.8%), the mortality rate was favorable at 2.3%-13.2%, suggesting the widespread use of surgical treatment and VA-ECMO [9]. In 2025, the Japanese Circulation Society published guidelines for PE, deep vein thrombosis (DVT), and pulmonary hypertension [10]. These guidelines recommend pulmonary artery thrombectomy (using cardiopulmonary bypass) as a Class I procedure for high-risk PE with a thrombus in the central pulmonary artery (pulmonary artery trunk and main pulmonary artery) in patients with contraindications to thrombolytic therapy, in whom thrombolytic therapy has been unsuccessful, in whom percutaneous extracorporeal circulation has been initiated, and in whom it is difficult to maintain hemodynamics with the administration of vasopressors. In addition, taxi drivers are prone to DVT and PE because they drive in the same position for long periods [11]. However, there are only a limited number of case reports on taxi drivers who develop PE and progress to shock while working [12,13].

In this report, we present the case of a 51-year-old male taxi driver who experienced shock due to acute PE. The patient was placed on VA-ECMO and eventually underwent SE, which saved his life. The clinical course of this case is highly instructive and may serve as a reference for other clinicians when they encounter similar patients in their treatment strategy.

## Case presentation

A 51-year-old taxi driver with a history of myocardial infarction suddenly experienced shortness of breath while working, collapsed, and was transported to our hospital by ambulance. He had a history of myocardial infarction, but had not visited a hospital or received any prescriptions in the past three years. At the time of admission, his vital signs indicated shock, with a blood pressure (BP) of 99/55 mmHg, heart rate of 129 beats/min, and respiratory rate of over 30 breaths/min.

Arterial blood gas analysis with an O_2_ 10 L reservoir mask revealed hypoxemia and acidosis (Table 1). This patient was classified as Class V, with a very high mortality risk, a PESI of 151 points, and a high-risk sPESI of 3 points.

**Table 1:** Laboratory tests ALP, alkaline phosphatase; ALT, alanine aminotransferase; APTT, activated partial thromboplastin time; AST, aspartate aminotransferase; FDP-DD, fibrinogen/fibrin degradation products-D dimer; γ-GTP, γ-glutamyl transpeptidase; LDH, lactate dehydrogenase; NT-proBNP, N-terminal fragment of pro-B-type natriuretic peptide; PT, prothrombin time; TSH, thyroid-stimulating hormone

Arterial blood gas	Value	Unit	Reference range
pH	7.172	-	7.35-7.45
pCO_2_	43.7	mmHg	35-45
pO_2_	32.8	mmHg	80-100
Complete blood count			
				White blood cells	12900	/μL	3300-8600
Red blood cells	504	×10^4^/μL	386-492
Hemoglobin	15.6	g/dL	11.6-14.8
Platelets	15.5	×10^4^/μL	15.8-34.8
Electrolytes			
				Sodium	142	mEq/L	138-145
Potassium	3.3	mEq/L	3.6-4.8
Chloride	103	mEq/L	101-108
Calcium	8.8	mg/dL	8.8-10.1
Inorganic phosphorus	7.5	mg/dL	2.7-4.6
Proteins, enzymes, and others			
				Blood urea nitrogen	13	mg/dL	8-20
Creatinine	1.63	mg/dL	0.46-0.79
Uric acid	9.5	mg/dL	3.7-7.8
Total bilirubin	0.7	mg/dL	0.4-1.5
AST	37	IU/L	13-30
ALT	30	IU/L	7-23
ALP	129	U/L	106-322
LDH	251	IU/L	119-229
γ-GTP	25	U/L	13-64
Total protein	6.8	g/dL	6.6-8.1
Albumin	3.4	g/dL	4.1-5.1
Blood glucose	309	mg/dL	73-109
Creatine kinase	55	IU/L	59-248
Troponin I	152.5	pg/mL	0-26.2
NT-proBNP	1986	pg/mL	0-125
C-reactive protein	2.89	mg/dL	<0.14
TSH	3.65	mIU/L	0.61-4.23
Free T4	1.09	ng/mL	0.70-1.48
Lactate	11.3	mmol/L	<2.0
Coagulation			
				APTT	27.5	sec	23.8-34.4
PT	10.9	sec	9.6-12.3
PT-INR	0.9	-	0-3.99
FDP-DD	26.7	μg/mL	0-0.5

**Figure 1:**
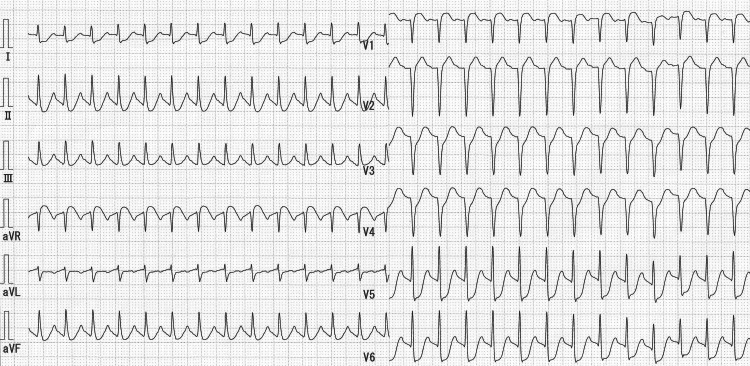
Electrocardiogram on admission Sinus tachycardia, ST-segment depression in leads I, II, aVL, aVF, and V3 to V6, and ST-segment elevation in lead aVR were observed.

Electrocardiography (ECG) showed sinus tachycardia; ST-segment depression in leads I, II, aVL, aVF, and V3 to V6; and ST-segment elevation in aVR (Figure 1).

**Figure 2:**
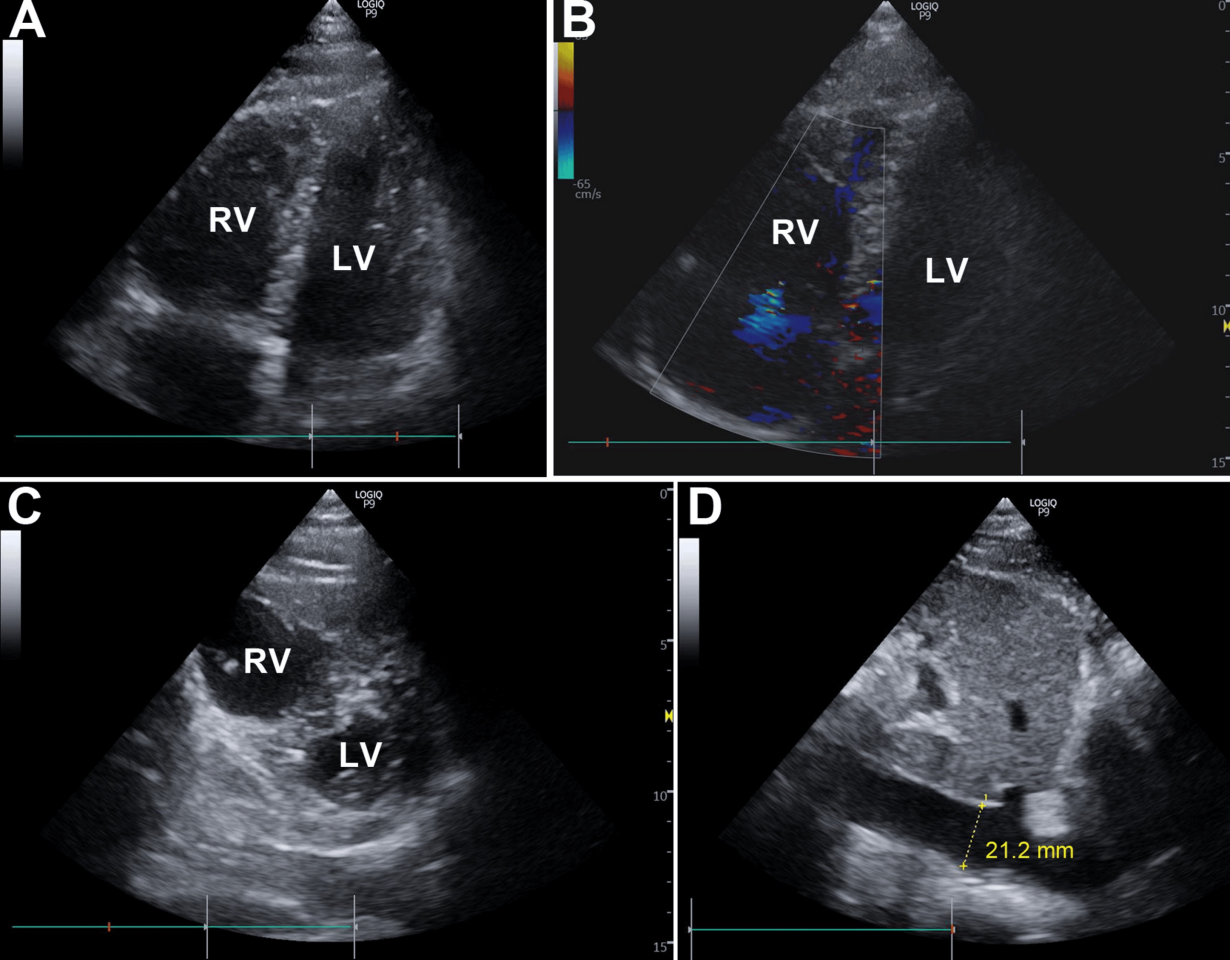
Echocardiography on admission Echocardiography demonstrates an enlarged RV (A), mild tricuspid regurgitation (B), D-sign formation in the LV (C), and poor respiratory variation of the inferior vena cava (D). LV, left ventricle; RV, right ventricle

Echocardiography revealed an enlarged right ventricle (RV), mild tricuspid regurgitation, D-sign formation in the left ventricle (LV), poor respiratory variation in the inferior vena cava (IVC), and apical hypokinesis of left ventricular systolic function (Figure 2). The fibrinogen/fibrin degradation products-D dimer (FDP-DD), troponin I (TnI), N-terminal fragment of pro-B-type natriuretic peptide (NT-proBNP), and lactate were elevated (Table 1). The patient was intubated, and dobutamine and norepinephrine were administered; however, hemodynamics could not be maintained. An intra-aortic balloon pump (IABP) was inserted along with heparin. Coronary angiography (CAG) revealed no significant stenosis, but a Swan-Ganz catheter revealed pulmonary hypertension (68/26 mmHg) in the main pulmonary artery.

**Figure 3:**
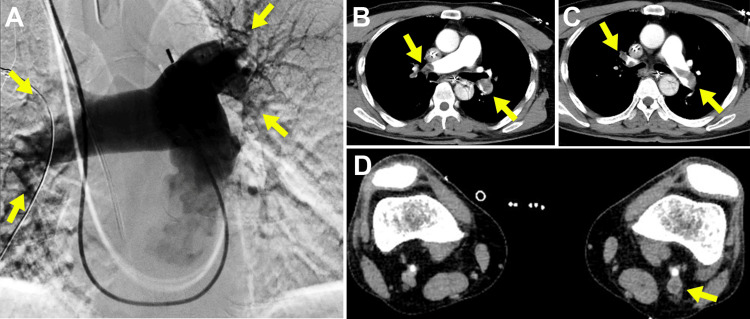
Pulmonary angiography Pulmonary angiography reveals bilateral pulmonary artery occlusion (A). Contrast-enhanced computed tomography reveals pulmonary embolism (B, C) and venous thrombosis in the left popliteal vein (D). Arrows indicate blood vessels occluded by thrombus.

Pulmonary angiography revealed bilateral pulmonary artery occlusion (Figure 3A), and the patient was diagnosed with acute PE. Contrast-enhanced computed tomography (CT) revealed PE and DVT (Figures 3B-3D), and continuous heparin was administered. The BP temporarily improved to 108/52 mmHg, and he was transferred to the Intensive Care Unit (ICU). However, one hour later, the hemodynamic status collapsed again, and the patient went into shock, necessitating CPR. VA-ECMO was promptly initiated within 10 minutes because femoral arterial and venous sheaths were placed prior to induction. Shortly thereafter, SE was performed on both pulmonary arteries. The postoperative course was relatively uneventful; the VA-ECMO was removed on the fourth day, the IABP was removed, and an IVC filter was placed on the fifth day. Thereafter, rivaroxaban was prescribed instead of heparin. On the 11th day of hospitalization, an increase in pericardial fluid was noted, necessitating temporary pericardial drainage, which was successfully removed three days later. The patient continued cardiac rehabilitation and was discharged with independent ambulation on the 30th day of hospitalization. At the time of discharge, the NT-proBNP level had decreased to 478 pg/mL, while the FDP-DD level had decreased to 1.2 μg/mL.

## Discussion

Acute PE remains a major clinical challenge, particularly in high-risk patients. Most risk models define high-risk patients based on hemodynamic instability, including shock or hypotension. However, systemic hypotension is typically a late marker of PE severity because PE primarily affects the RV while sparing the LV [9]. Therefore, systemic BP alone may not be a reliable determinant of clinical stability. Further risk stratification is usually based on age, major clinical parameters, and comorbidities, as well as PESI, sPESI, cardiac biomarkers (troponin and NT-proBNP), and imaging signs of RV dysfunction using echocardiography and/or CT [3,10,14]. Our patient presented with hypotension, high PESI and sPESI scores, elevated TnI and NT-proBNP levels, and RV dysfunction on echocardiography. All indicators placed the patient in the high-risk group, and hemodynamic maintenance and reperfusion therapies were recommended. In this case, myocardial infarction involving the left main coronary artery (LMCA) was initially suspected, and it was necessary to at least promptly rule it out. Therefore, an IABP was inserted, and CAG was performed. As the patient’s hemodynamic status temporarily improved thereafter, we opted to defer VA-ECMO initiation. Moreover, in anticipation of future surgical intervention, thrombolytic therapy was not administered immediately, although our patient did not have a contraindication for thrombolytic therapy, and an aspiration catheter system was not available in our hospital.

The role of SE in managing high-risk PE may be underestimated in current clinical practice [15,16]. According to the Japanese Cardiovascular Surgery Database, preoperative CPR was required in 27.6% of patients, and preoperative VA-ECMO was performed in 26.5% of patients with acute PE. Urgent or emergency operations were performed in 93% of patients. The operative mortality rate was 73/355 (20.6%) [17]. The outcome of SE was acceptable, considering the urgency of the situation and the preoperative comorbidities of the patients. Moreover, failure of systemic thrombolytic therapy, or the use of systemic thrombolytics after ECMO insertion, was associated with increased mortality and bleeding complications [18,19]. Our patient was initially monitored in the ICU with IABP and continuous heparin administration. However, the patient’s hemodynamics collapsed again; therefore, VA-ECMO was initiated. SE was performed relatively quickly after the insertion of VA-ECMO. Therefore, the patient did not develop clinically significant bleeding complications.

In a previous report, patients working as taxi drivers presented with high frequencies of organized thrombi in the pulmonary arteries [11]. These results suggest that prolonged venous congestion in the legs increases the risk of DVT and PE. In fact, our patient had been working for 14 hours on the day he developed PE and DVT in the lower extremities.

Interestingly, the ECG taken at the time of admission suggested an LMCA infarction. There have been previous reports of patients with PE presenting with ECG findings suggestive of LMCA infarction; such an ECG can occur when shock causes generalized myocardial ischemia [12,20].

## Conclusions

In high-risk PE, immediate hemodynamic stabilization, consideration of alternative causes of shock - such as LMCA infarction - and prompt initiation of appropriate reperfusion therapy are critical to improving outcomes.
